# Distinct single cell signal transduction signatures in leukocyte subsets stimulated with khat extract, amphetamine-like cathinone, cathine or norephedrine

**DOI:** 10.1186/2050-6511-14-35

**Published:** 2013-07-11

**Authors:** Therese Bredholt, Elisabeth Ersvær, Bjarte Skoe Erikstein, André Sulen, Håkon Reikvam, Hans Jørgen Aarstad, Anne Christine Johannessen, Olav Karsten Vintermyr, Øystein Bruserud, Bjørn Tore Gjertsen

**Affiliations:** 1 Department of Clinical Science, University of Bergen, Bergen, Norway; 2 Institute of Biomedical Laboratory Sciences, Bergen University College, Bergen, Norway; 3 Department of Internal Medicine, Haukeland University Hospital, Bergen, Norway; 4 Department of Head and Neck Surgery, Haukeland University Hospital, Bergen, Norway; 5 Department of Clinical Medicine, University of Bergen, Bergen, Norway; 6 Department of Pathology, Haukeland University Hospital, Bergen, Norway

**Keywords:** Signal transduction, Leukocytes, Single-cell modification-specific flow cytometry, Natural amphetamine, Cathinone, Khat-extract

## Abstract

**Background:**

Amphetamine and amphetamine derivatives are suggested to induce an immunosuppressive effect. However, knowledge of how amphetamines modulate intracellular signaling pathways in cells of the immune system is limited. We have studied phosphorylation of signal transduction proteins (Akt, CREB, ERK1/2, NF-κB, c-Cbl, STAT1/3/5/6) and stress sensors (p38 MAPK, p53) in human leukocyte subsets following *in vitro* treatment with the natural amphetamine cathinone, the cathinone derivatives cathine and norephedrine, in comparison with a defined extract of the psychostimulating herb khat (*Catha edulis* Forsk.). Intracellular protein modifications in single cells were studied using immunostaining and flow cytometry, cell viability was determined by Annexin V-FITC/Propidium Iodide staining, and T-lymphocyte proliferation was measured by ^3^H-thymidine incorporation.

**Results:**

Cathinone, cathine and norephedrine generally reduced post-translational modifications of intracellular signal transducers in T-lymphocytes, B-lymphocytes, natural killer cells and monocytes, most prominently affecting c-Cbl (pTyr700), ERK1/2 (p-Thr202/p-Tyr204), p38 MAPK (p-Thr180/p-Tyr182) and p53 (both total p53 protein and p-Ser15). In contrast, the botanical khat-extract induced protein phosphorylation of STAT1 (p-Tyr701), STAT6 (p-Tyr641), c-Cbl (pTyr700), ERK1/2 (p-Thr202/p-Tyr204), NF-κB (p-Ser529), Akt (p-Ser473), p38 MAPK (p-Thr180/p-Tyr182), p53 (Ser15) as well as total p53 protein. Cathinone, cathine and norephedrine resulted in unique signaling profiles, with B-lymphocytes and natural killer cells more responsive compared to T-lymphocytes and monocytes. Treatment with norephedrine resulted in significantly increased T-lymphocyte proliferation, whereas khat-extract reduced proliferation and induced cell death.

**Conclusions:**

Single-cell signal transduction analyses of leukocytes distinctively discriminated between stimulation with cathinone and the structurally similar derivatives cathine and norephedrine. Cathinone, cathine and norephedrine reduced phosphorylation of c-Cbl, ERK1/2, p38 MAPK and p53(Ser15), and norephedrine induced T-lymphocyte proliferation. Khat-extract induced protein phosphorylation of signal transducers, p38 MAPK and p53, followed by reduced cell proliferation and cell death. This study suggests that protein modification-specific single-cell analysis of immune cells could unravel pharmacologic effects of amphetamines and amphetamine-like agents, and further could represent a valuable tool in elucidation of mechanism(s) of action of complex botanical extracts.

## Background

The herbal drug khat (*Catha edulis* Forsk.) is chewed for its psychostimulating effects by millions of people [1,2]. The alkaloid cathinone represents its main psychoactive compound, with a structure and pharmacological profile similar to the synthetic drug amphetamine [3]. Cathinone represents a labile precursor for cathine and norephedrine [3], being metabolized primarily to norephedrine *in vivo*[4]. In addition to cathinone and its derivates, khat contains other bioactive constituents, including tannins, ascorbic acid, the phenylalkylamine meruchatine [5], catheduline alkaloids [6], flavonoids and triterpenoids [3,7].

During khat chewing, cathinone, cathine and norephedrine are extracted into saliva, and the oral buccal mucosa plays a major role in absorption [8]. Like amphetamine, cathinone induces release of dopamine, serotonin (5-hydroxytryptamine, 5-HT) and noradrenaline from pre-synaptic neuronal terminals, and has further been suggested to inhibit neurotransmitter reuptake [3]. Cathinone, cathine and norephedrine have been shown to act as substrates at the noradrenaline and dopamine transporters, to bind α_2_-adrenergic receptors [9,10], and cathinone has in addition been reported to bind 5-HT receptors [10,11]. The β_1_-adrenergic receptor was suggested to mediate khat-induced increase in systolic blood pressure and pulse rate [12], whereas the α_1_-adrenergic receptor has been implicated in bladder dysfunction following khat chewing [13].

Leukocytes express transporters and receptors for dopamine and serotonin, and several types of immune cells store these neurotransmitters (reviewed in [14,15]). It has further been suggested that lymphocytes are capable of producing dopamine [16,17]. Leukocytes are known to express adrenergic receptors, and expression of the noradrenaline transporter has also been reported [18]. Various drugs that influence neurotransmitter transport, including anti-depressants and amphetamines, have demonstrated immunosuppressive effects *in vivo* (reviewed in [14,19]). Several studies have suggested that at least part of the immunosuppression could be due to direct impact of such pharmacological substrates on immune cells. For instance, House et al. reported that amphetamine could suppress B-lymphocyte proliferation and IL-2 secretion from T-lymphocytes *in vitro*[20]. Amphetamine was further seen to reduce zymosan-induced phagocytosis both *in vitro* and *in vivo*[21].

Intracellular signal transduction proteins and cell stress sensors have been linked to activation of adrenergic, dopaminergic and serotonergic receptors, which represent G-protein coupled receptors. The β-adrenergic/G stimulatory/protein kinase A pathway was reported to induce phosphorylation of mitogen-activated protein kinase (MAPK) p38 in activated B-lymphocytes [22] and in T-lineage cells [23]. 5-HT_7_ receptor stimulation on naïve T-lymphocytes has been suggested to induce activation of extra-cellular regulated kinase 1/2 (ERK1/2) [24]. Further, stimulation of dopaminergic D2-like receptors was reported to reduce phosphorylation of cyclic AMP response element binding (CREB) protein in T-lymphocytes [25].

In this study we exposed peripheral blood leukocytes *in vitro* to cathinone, cathine, norephedrine, and to physiological relevant concentrations of khat-extract, and investigated the impact on selected signaling and stress sensor proteins. The distinct signatures of protein modifications detected in the leukocyte subsets indicate cellular effects and mechanism(s) of action of the khat-derived alkaloids, and further demonstrate that the cellular signaling response diverge from treatment with a complex botanical khat-extract.

## Results

**Figure 1:**
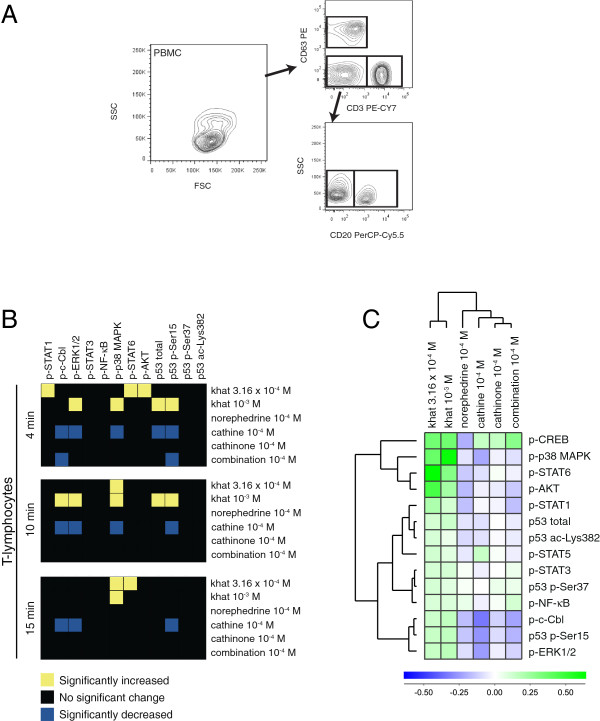
**Gating strategies and signal transduction in T-lymphocytes.** PBMCs (*n = 6*) were stimulated with khat-extract (10^-3^ and 3.16 × 10^-4^ dilutions), cathinone, cathine, norephedrine (10^-4^ M) and a combination of cathinone and norephedrine (combination: 2 × 10^-4^ M) for 4, 10 and 15 minutes, *in vitro*. **(A)** The PBMCs were fixed, permeabilized, stained with antibodies against specific cell surface markers and modification-specific antibodies and analyzed by flow cytometry. The PBMCs were identified as T-lymphocytes (CD3^+^ CD20^-^ CD163^-^), B-lymphocytes (CD3^-^ CD20^+^ CD163^-^), NK cells (CD3^-^ CD20^-^ CD163^-^) and monocytes (CD3^-^ CD20^-^ CD163^+^). **(B)** Heatmaps of statistical significant changes in protein-modification levels and total protein levels in T-lymphocytes. The analysis is based on fold change values for the modification-specific antibodies calculated from MFI measurements: MFI_stimulated_/MFI_control_. ANOVA of the mean was used to determine statistical significant changes. **(C)** Unsupervised hierarchical clustering analysis of the different stimuli and signal transduction proteins in T-lymphocytes following 15 minutes of stimulation. The mean fold changes were calculated, and log(2) converted before the unsupervised hierarchical clustering analysis was performed.

Peripheral blood mononuclear cells (PBMCs) from healthy donors (*n = 6*) were treated with khat-extract (10^-3^ and 3.16 × 10^-4^ dilutions), cathinone, cathine and norephedrine (10^-4^ M concentrations) for 4, 10 and 15 minutes, *in vitro*. Based on cathinone, cathine and norephedrine concentrations in the khat-extract, the experimental dilutions should be comparative to local concentrations of khat constituents *in vivo* during khat chewing (Table 1) [26]. The impact on signal transduction proteins and stress sensors in T-lymphocytes, B-lymphocytes, natural killer (NK) cells and monocytes was assessed using modification-specific antibodies and flow cytometric analyses (Figure 1A).

**Table 1:** Cathinone, cathine and norephedrine concentrations in the khat-extract stock solution, and in experimental cell cultures

	**Cathinone**		**Cathine**		**Norephedrine**	
	**Mw 150.1**		**Mw 152.1**		**Mw 152.1**	
	**mg ml**^**-1**^	**M**	**mg ml**^**-1**^	**M**	**mg ml**^**-1**^	**M**
khat-extract stock solution	2.5	1.7 × 10^-2^	3.0	2.0 × 10^-2^	0.3	2.0 × 10^-3^
10^-3^ dilution	2.5 × 10^-3^	1.7 × 10^-5^	3 × 10^-3^	2.0 × 10^-5^	3 × 10^-4^	2.0 × 10^-6^
3.16 × 10^-4^ dilution	7.9 × 10^-4^	5.4 × 10^-6^	9.5 × 10^-4^	6.3 × 10^-6^	9.5 × 10^-5^	6.3 × 10^-7^

The stock solution concentrations were determined based on HPLC-MS-MS analyses [30].

**Figure 2:**
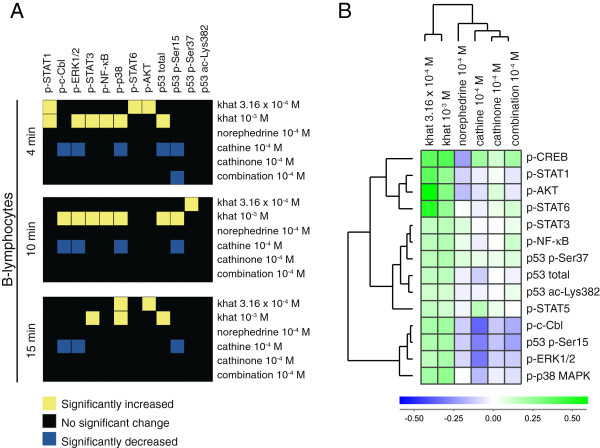
**Signal transduction in B-lymphocytes. (A)** Heatmap of statistical significant changes in protein-modification levels and total protein levels in B-lymphocytes. **(B)** Unsupervised hierarchical clustering analysis of the different stimuli and signal transduction proteins in B-lymphocytes following 15 minutes of stimulation.

**Figure 3:**
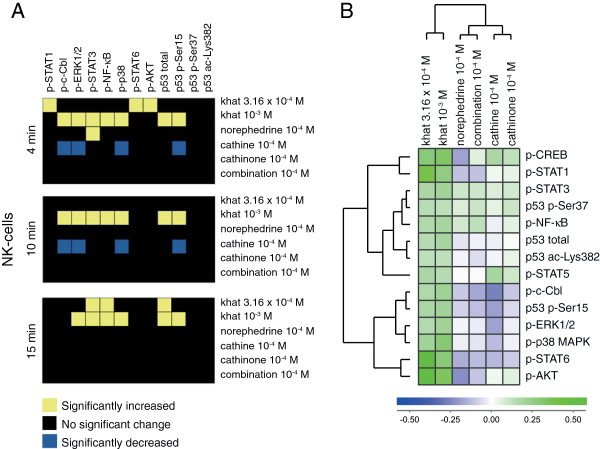
**Signal transduction in NK cells. (A)** Heatmap of statistical significant changes in protein-modification levels and total protein levels in NK-cells. **(B)** Unsupervised hierarchical clustering analysis of the different stimuli and signal transduction proteins in NK-cells following 15 minutes of stimulation.

**Figure 4:**
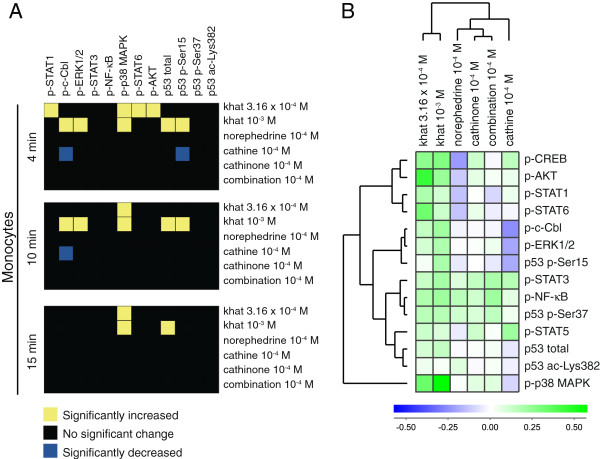
**Signal transduction in monocytes. (A)** Heatmap of statistical significant changes in protein-modification levels and total protein levels in monocytes. **(B)** Unsupervised hierarchical clustering analysis of the different stimuli and signal transduction proteins in monocytes following 15 minutes of stimulation.

Fold change values based on median fluorescence intensity (MFI) measurements were used as basis for data analyses, and signal transduction signatures for the leukocyte subsets are displayed as heatmaps of statistical significant changes (Figure 1B, 2A, 3A, 4A), and as cluster analyses (Figure 1C, 2B, 3B, 4B). The levels of phosphorylated (p) signal transducer and activator of transcription (STAT) 5, p-CREB, and p53 acetylated (ac)-Lysine (Lys)382 were not significantly altered by any stimulus (Additional file 1: Table S1) and were excluded from the heatmaps (Figure 1B, 2A, 3A, 4A) but included in the cluster analyses of signaling responses (Figure 1C, 2B, 3B, 4B). Cathinone did not induce significant signaling responses at the concentration and exposure times tested.

### Signal transduction in T-lymphocytes

For T-lymphocytes (Figure 1B), treatment with the lowest khat-extract concentration induced p-STAT1, p-p38 MAPK, p-STAT6 and p-AKT; the highest khat-extract concentration induced p-c-Cbl, p-ERK1/2, p-p38 MAPK, p53 p-Serine (Ser)15 and total p53; cathine reduced p-c-Cbl, p-ERK1/2, p-p38 MAPK, p53 p-Ser15 and total p53; the combination norephedrine/cathinone reduced p-c-Cbl and p53 p-Ser15. The time-points and p-values for these changes are listed in Additional file 1: Table S1.

The cluster analysis of signal transduction in T-lymphocytes following 15 minutes of stimulation present significant and non-significant changes (Figure 1C). Khat-extract generally increased protein phosphorylation, in contrast to cathinone, cathine and norephedrine, which reduced phosphorylation of several proteins. Cathinone and its derivatives clustered separately from khat-extract, indicating different pharmacological properties of the complex extract compared to the pure khat components.

### Signal transduction in B-lymphocytes

For B-lymphocytes (Figure 2A), treatment with the lowest khat-extract concentration induced p-STAT1, p-p38 MAPK, p-STAT6, p-AKT and p53 p-Ser37; the highest concentration of khat-extract induced p-STAT1, p-c-Cbl, p-ERK1/2, p-STAT3, p-NF-κB, p-p38 MAPK, p53 p-Ser15 and total p53. In contrast, cathine reduced p-c-Cbl, p-ERK1/2, p-p38 MAPK, p53 p-Ser15 and total p53; the combination norephedrine/cathinone reduced p53 p-Ser15. The time-points and p-values for these changes are listed in Additional file 1: Table S1.

The cluster analysis of signal transduction in B-lymphocytes (15 minutes) displays significant and non-significant changes (Figure 2B). As in T-lymphocytes, khat-extract induced protein phosphorylation, whereas cathinone and its derivatives decreased phosphorylation levels. In particular, p-c-Cbl, p-ERK1/2, p-p38- MAPK and p53 p-Ser15 were prominently reduced, and these phosphorylation responses clustered together.

### Signal transduction in natural killer cells

For NK cells (Figure 3A), treatment with the lowest khat-extract concentration induced p-STAT1, p-STAT3, p-NF-κB, p-STAT6, p-AKT and total p53; the highest khat-extract concentration induced p-c-Cbl, p-ERK1/2, p-STAT3, p-NF-κB, p-p38 MAPK, p53 p-Ser15 and total p53; norephedrine induced p-STAT3; cathine reduced p-c-Cbl, p-ERK1/2, p-p38 MAPK and p53 p-Ser15. The time-points and p-values for these changes are listed in Additional file 1: Table S1. The cluster analysis of signal transduction in NK cells (15 minutes) displays significant and non-significant changes (Figure 3B). In NK cells, cathinone and its derivatives both induced and decreased protein phosphorylation.

### Signal transduction in monocytes

For monocytes (Figure 4A), treatment with the lowest khat-extract concentration induced p-STAT1, p-p38 MAPK, p-STAT6 and p-AKT; the highest khat-extract concentration induced p-c-Cbl, p-ERK1/2, p-p38 MAPK, p53 p-Ser15 and total p53; cathine reduced p-c-Cbl and p53 p-Ser15. The cluster analysis of signal transduction in monocytes (15 minutes) indicates significant and non-significant changes (Figure 4B). Cathinone and its derivatives gave a less pronounced reduction in phosphorylation levels in the monocyte subset.

### Alterations in p53 post-translational modifications

**Figure 5:**
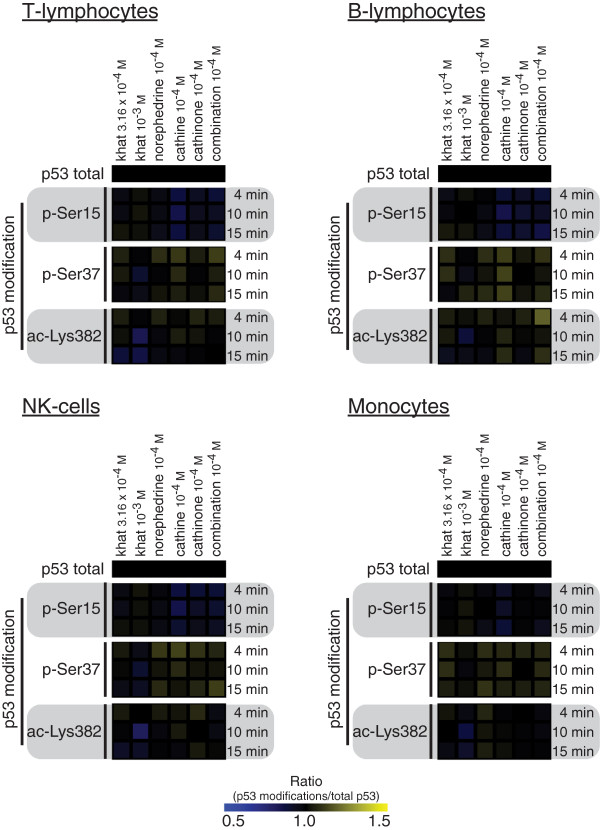
**p53 post-translational modifications relative to total p53.** Experiments and flow cytometry analyses as in the legend to Figure 1. Based on MFI values the median ratio of p-Ser15, p-Ser37 and ac-Lys382 relative to total p53 levels were determined for the various stimuli and time-points for the different leukocyte subsets.

Based on MFI values for total p53, p53 p-Ser15, p53 p-Ser37 and p53 ac-Lys382, fold change values of p53 post-translational modifications relative to total p53 level were calculated (Figure 5). Significant changes in phosphorylation/acetylation of p53 were seen for all leukocyte subsets. For details on time-points and p-values, see Additional file 2: Table S2.

For T-lymphocytes, the highest khat-extract concentration reduced p-Ser37; cathine, cathinone and the combination norephedrine/cathinone reduced p-Ser15. For B-lymphocytes, cathine, cathinone and norephedrine/cathinone reduced p-Ser15, while cathine increased p-Ser37. For NK cells, the highest khat-extract concentration reduced p-Ser37 and ac-Lys382; cathine, cathinone and norephedrine/cathinone all reduced p-Ser15. In monocytes, the highest khat-extract concentration reduced ac-Lys382; cathine reduced p-Ser15. The observations of modifications of central signal transduction pathways and stress proteins were extended with analyses of effects on cell death and proliferation in an attempt to connect early signal transduction responses with later functional effects on leukocytes.

### Khat, cathinone and its derivatives affected leukocyte viability and proliferation

**Figure 6:**
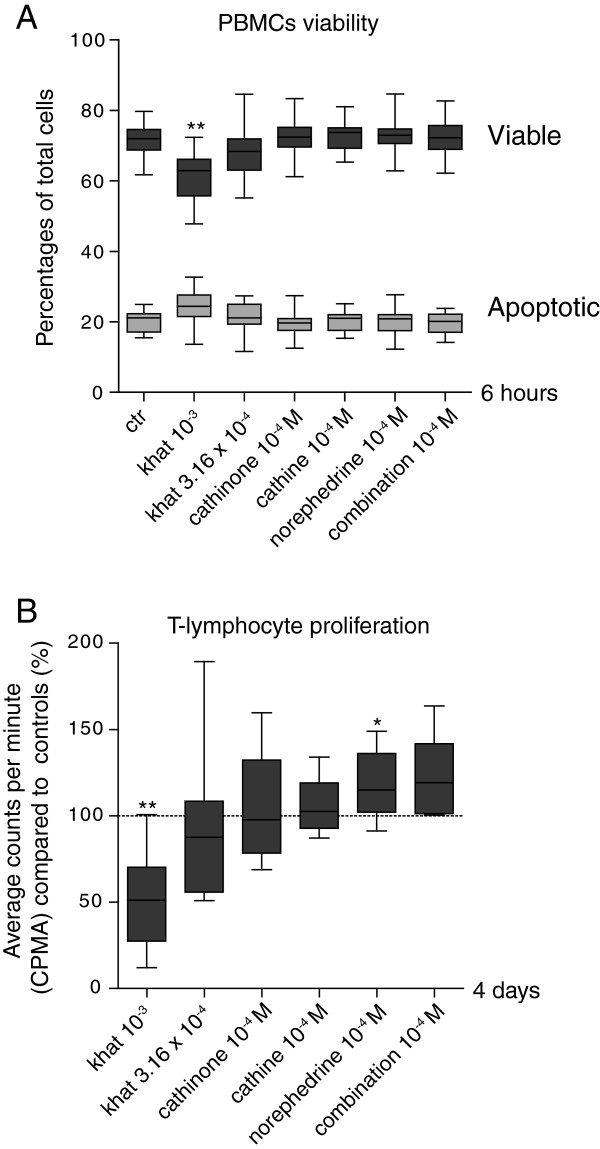
**Functional effects: cell death and T-lymphocyte proliferation. (A)** PBMCs were treated with khat (k 10^-3^ and 3.16 × 10^-4^ dilutions), 10^-4^ M of cathinone, cathine, norephedrine (*n = 14*), and a combination of the latter three (*n = 7*) for 6 hours. The PBMCs were stained with Annexin V-FITC and PI, and analyzed by flow cytometry. The results are expressed as percentages viable (Annexin V^-^PI^-^) and apoptotic (Annexin V^+^PI^-^) cells of the total cell number analyzed. **(B)** PBMCs were treated with khat (10^-3^ and 3.16 × 10^-4^ dilutions) and 10^-4^ M of cathinone, cathine, norephedrine (*n = 14*) and a combination of the latter three (*n = 7*). Nuclear radioactivity following ^3^H-thymidine incorporation was assayed by liquid scintillation counting after four days. Results are expressed as percent average counts per minute (CPMA) in treated cells relative to the CPMA in control cells. * denotes p <0.008 and ** denotes p <0.001.

Levels of apoptosis were determined after 6 hours of stimulation, using Annexin V-fluorescein isothiocyanate **(**FITC) and propidium iodide (PI) staining followed by flow cytometric analyses (Figure 6A). The highest khat-extract concentration significantly reduced the levels of viable PBMCs, whereas the lower khat-extract concentration, cathinone, cathine and norephedrine had no significant effects.

Effects on T-lymphocyte proliferation were assessed following 4 days of stimulation, based on ^3^H-thymidine incorporation (Figure 6B). The highest khat-extract concentration inhibited proliferation by approximately 50%. Treatment with cathinone and cathine gave elevated CPMA values, and norephedrine significantly increased T-lymphocyte proliferation (p < 0.008).

## Discussion

**Figure 7:**
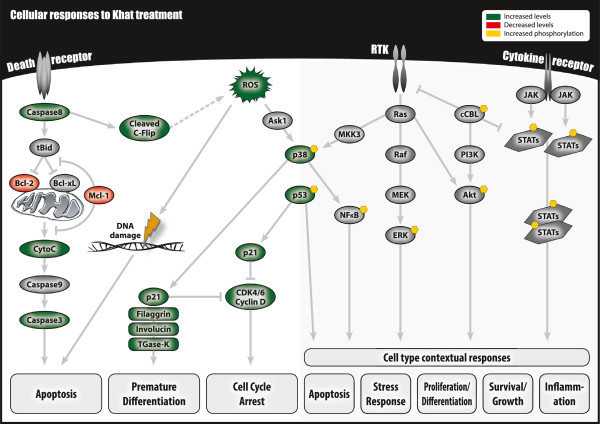
**A summary of previous and present findings of cellular effects of khat-extract.** Our studies have shown that exposure of various cell types (i.e. leukemic cell lines, normal oral keratinocytes and fibroblasts, normal peripheral leukocytes) to khat-extract *in vitro* affects various proteins involved in cell death, cell stress, cell cycle regulation, differentiation and cell signaling [26,30,31,33,37].

**Figure 8:**
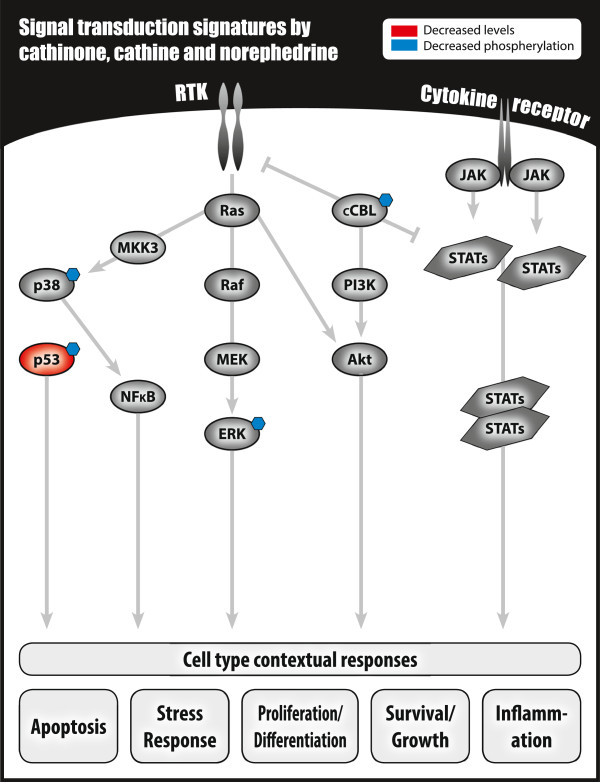
A summary of our present findings of cell signal transduction signatures in leukocytes stimulated with amphetamine-like cathinone, cathine or norephedrine.

Single-cell profiling of signal transduction using modification-specific antibodies and flow cytometry represents a powerful method in analyses of signaling networks [27-29]. Here we used this method to investigate the impact on signal transduction in leukocyte subsets following short-time exposure (4, 10 and 15 minutes) to the natural khat-derived amphetamine-like cathinone, its derivatives cathine and norephedrine, and a partly characterized khat-extract, *in vitro*. Functional implications were assessed based on determination of cell death induction and T-lymphocyte proliferation following long-time exposure (4 days). A summary of our present and previous findings regarding in vitro cellular effects of khat-extract is presented in Figure 7, and the present findings regarding cathinone and its natural derivatives is presented in Figure 8.

Cathinone and its natural derivatives generally suppressed basal phosphorylation of the examined signal transducers and stress sensors, while khat-extract induced protein post-translational modifications (Figures 1, 2, 3 and 4). The different impact on signal transduction proteins in the leukocytes was reflected in the functional assays, demonstrating khat-induced cell death and reduced T-lymphocyte proliferation, whereas no cytotoxic effects were detected for cathinone, cathine and norephedrine (Figure 6). Toxic effects by khat have been reported in *in vivo* and *in vitro* studies [3,30-32]. We have previously shown that khat induce caspase-dependent apoptosis in myeloid cells and keratinocytes [30,31,33]. Khat-induced apoptosis may involve induction of reactive oxygen species [31] (see also Aleruyani et al. for review [34]), which is described to function upstream of p53 and p38 in some model systems, while in others ROS production could be a downstream effect of p53 activation [35,36]. We therefore anticipated that single cell signalling analysis of total and modified p53 and phosphorylated p38 MAPK should reveal the mechanisms involved in khat toxicity when compared with cathinone and its natural derivatives.

There are conflicting results regarding toxic effects by cathinone and its natural derivatives [20,26,33]. Here we observed that norephedrine significantly increased T-lymphocyte proliferation after long-time exposure. A study by House and co-workers, comparing synthetic amphetamines and cathinone, demonstrated that cathinone induced B-lymphocyte proliferation, cytotoxic T-lymphocyte induction and IL-2 production [20]. As far as we know, a cell proliferative effect by norephedrine has not previously been demonstrated *in vitro*.

We have previously reported that khat-extract induced the tumor suppressor and cellular stress sensor p53 in normal oral keratinocytes and fibroblasts [26]. Here we demonstrate that p53 protein accumulates in leukocytes following khat-extract treatment, accompanied by, in particular, increased Ser15 phosphorylation (Figures 1, 2, 3 and 4). In contrast to khat-extract, cathinone and its derivatives generally reduced total p53 levels, p53 Ser15 phosphorylation (Figures 1, 2, 3 and 4) and Ser15 phosphorylation was further seen reduced relative to total p53 (Figure 5).

Cathine significantly reduced p38 MAPK phosphorylation in the various leukocyte subsets, whereas khat-extract mediated a significant increase in phosphorylation of this cell stress protein. In agreement with our observations, khat was recently reported to induce p38 MAPK phosphorylation in PBMCs *in vitro*, and khat-treatment was seen to induce cell death by apoptosis [32]. p38 is probably acting upstream of p53, and may be pivotal in khat-induced abnormal differentiation of *in vitro*-reconstructed human normal buccal mucosa [37]. Additionally, our data indicate that in particular cathine attenuated c-Cbl, ERK1/2 and p53 Ser15 phosphorylation in the various subsets, further including p-STAT6 and p-Akt reduction in T-lymphocytes (Figure 1). Interestingly, c-Cbl was recently reported to play an important role in trafficking of the 5-HT_2A_ receptor in human embryonic kidney cells [38], whereas de-phosphorylation of AKT was observed following stimulation of striatal class D2 dopamine receptors [39,40].

Our results suggest that cathinone and its derivatives generally reduced basal phosphorylation levels of the proteins analysed, but as an exception, norephedrine was seen to significantly induce phosphorylation of STAT3 in NK cells. STAT3 is known to induce both proliferative and anti-proliferative effects, but a recent study suggested that STAT3 could mediate an immunosuppressive effect in NK cells [41]. Further, even if not statistical significant, cathine and cathinone indicated elevated p-CREB levels in all leukocyte subsets, whereas norephedrine reduced basal phosphorylation of CREB. Our results may indicate that the alkaloids cathine and cathinone induce CREB phosphorylation as has been shown for e.g. ephedrine. Ephedrine, an alkaloid chemically similar to cathinone, has been suggested to induce PKA-mediated CREB phosphorylation through β-adrenergic receptor/cyclic AMP/PKA/DARPP-32 signaling pathway [42]. Taken together these observations show that cathinone, cathine and norephedrine resulted in distinct signaling signatures, even if the compounds are closely related structurally.

The leukocyte subsets differed in their signaling responses to the various treatments, and B-lymphocytes and NK cells were generally seen as more responsive compared to T-lymphocytes and monocytes. This observation could relate to varying levels or different repertoires of monoamine transporters and receptors on the immune cells. For instance, T-lymphocytes and monocytes were reported to exhibit a low expression of dopamine receptors when compared to B-lymphocytes and NK cells, which displayed a higher and more consistent expression [43]. The mechanism(s) behind the different responses to khat-extract, cathinone and its derivatives seen in the various leukocyte subsets should be addressed in future studies.

In recent years, synthetic cathinone derivatives have been designed, being widely distributed as recreational drugs. The use of these drugs has been reported with harmful health effects, and has been suspected to be involved in several deaths [44]. Signal transduction studies and functional assays of synthetic cathinone derivatives and other amphetamine-like agents, like the ones performed here, could be useful in evaluation of potential harmful short term and long term effects on various immune cells. In this study we observed significant changes in phosphorylation levels for several of the investigated signal transduction and cell stress proteins, but the changes were still modest. In order to evaluate more fully the impact of pharmacological substrates on cells of the immune system, the approach should be extended and involve analyses of immune cells following *in vivo* studies with experimental animals.

## Conclusions

We report that the khat-derived cathinone and its derivatives generally reduced the levels of activating protein modifications of signal transducers and stress sensors in leukocytes, and that norephedrine increased *in vitro* T-lymphocyte proliferation. The closely structurally related compounds cathinone, cathine and norephedrine were seen to induce unique signaling signatures in leukocytes. Further, this study confirmed that compounds in a botanical khat-extract activated cell stress sensors in immune cells and induced cytotoxic effects. Our results suggest that single-cell flow cytometric analysis of intracellular signaling pathways may be an informative and feasible approach when elucidating pharmacological effects and mechanisms of action in immune cells.

## Methods

### Blood donors

Blood was obtained from healthy donors from Department of Immunology and Transfusion Medicine, Haukeland University Hospital, Bergen, Norway. The blood donors used in the signal transduction analyses were 6 healthy individuals (4 females, 2 males) with a mean age of 38.2 years (range: 24–59). The use of blood for research purposes was approved by the local ethics committee REK (Regionale komiteer for medisinsk og helsefaglig forskningsettikk), Haukeland University Hospital, and samples collected after written consent by the blood donors, in accordance with the Declaration of Helsinki.

### Cathinone, cathine and norephedrine

S-(−)-cathinone, (1S,2S)-(+)-norpseudoephedrine (cathine) and (1R,2S)-(−)-norephedrine were purchased from Sigma-Aldrich, and dissolved in DMSO (Sigma-Aldrich). These compounds were used in cell cultures at the concentration 10^-4^ M, and import and experiments were performed after written permission from The Norwegian Medicines Agency.

### Khat-extract

Khat (asili cultivate) from the Meru district in Kenya was purchased in Nairobi, transported at 4°C under moist conditions, and the material was extracted within 48 hrs of harvest with methanol [30,33]. The khat-extract was prepared from approximately 200 g of khat, using the leaves and the soft parts of the twigs, which were chopped into pieces of approximately 5 mm length and submerged in methanol. The methanol extract was dried using a rotary evaporator, the semi-solid residue dissolved at a concentration of 0.2 g/ml in dimethylsulphoxide (DMSO; Sigma, St. Louis, MO, USA) and the stock solution stored at −80°C.

The stock solution was diluted in Roswell Park Memorial Institute (RPMI)-1640 medium (Sigma-Aldrich) with 10% fetal bovine serum (FBS), 2 mM L-glutamine and antibiotics (100 IU ml^-1^ penicillin and 100 μg ml^-1^ streptomycin; Gibco, Grand Island, NY, USA), and precipitates removed by centrifugation (10 000 g, 15 minutes, 4°C) [30]. The khat-extract supernatant was added experimental cell cultures giving final dilutions of 10^-3^ and 3.16 × 10^-4^, based on previous studies [30]. All experimental cell cultures contained 0.1% DMSO.

Cathinone is relatively unstable, being transformed to cathine upon wilting of khat leaves, while being metabolized predominantly to norephedrine *in vivo*[3,4]. Cathinone, cathine and norephedrine are therefore suited as reference substances, indicating the freshness of the khat sample and the stability of the khat-extract. The concentrations of cathinone, cathine and norephedrine in the khat-extract were determined using high pressure liquid chromatography and mass spectrometry (HPLC-MS-MS), as previously described [30], and the values are displayed in Table 1, together with experimental cell culture concentrations.

In order to import and study khat, classified as an illegal narcotic substance in Norway, written permission was obtained from The Norwegian Medicines Agency (permission 01/705 of 2002-07-08).

### Peripheral blood mononuclear cells

Peripheral blood mononuclear cells (PBMCs) were isolated from buffy coats using density gradient separation (Lymphoprep; Nycomed, Oslo, Norway; specific density 1.077), and resuspended at: (i) 1 × 10^6^ cells ml^-1^ in X-vivo10® culture medium (BioWhittaker, Walkersville, MA, USA) with 100 μg ml^-1^ gentamicin for use in proliferation studies, (ii) 1 × 10^6^ cells ml^-1^ in RPMI-1640 medium (10% FBS, L-glutamine, antibiotics) for studies on signal transduction, or (iii) 200 000 cells ml^-1^ in RPMI-1640 medium (10% FBS, L-glutamine, antibiotics) when determining apoptosis.

### Annexin V-fluorescein isothiocyanate (FITC)/propidium iodide (PI) staining

When determining apoptosis, the PBMCs were stained with Annexin V-FITC and PI, using the Apoptest™ kit from Nexins Research (Hoeven, the Netherlands) according to the manufacturer’s protocol. Data were collected on a FACS Calibur flow cytometer (Becton Dickinson, BD, Immunocytometry Systems, San Jose, CA, USA) and analyzed with FlowJo 7.2.5 software (TreeStar Inc., Ashland, OR, USA).

### ^3^H-thymidine proliferation assay

The assay used to determine *in vitro* lymphocyte proliferation is based on a whole blood protocol [45]. Experiments were performed in triplicates in 96-well plates, and each well added (i) 5 × 10^4^ PBMCs; (ii) CD3 antibody (mouse IgE Moab; CLB-T3/4.E), final concentration 316 ng ml^-1^; (iii) CD28 antibody (mouse IgG1 Moab, CLB-CD28/1), final concentration 842 ng ml^-1^; and (iv) test compound (cathinone, cathine, norephedrine, khat-extract) or control medium. The cell cultures were incubated (37°C, 5% CO_2_) for 3 days, added ^3^H-thymidine (37 kBq per well; TRA 310, Amersham International, Amersham, UK), and nuclear radioactivity assayed on day four by liquid scintillation counting. Results are expressed as percent average counts per minute (CPMA) in treated cells relative to CPMA in controls: (CPMA_treated_/CPMA_control_) × 100. All CPMA values were >1000, indicating proliferating cells.

### Intracellular staining of proteins and flow cytometric analyses

PBMCs were fixed in 1.6% paraformaldehyde, permeabilized with 100% methanol and stored at −80°C until flow cytometric analysis. To achieve high-throughput and to reduce costs, the PBMCs were barcoded [29], and intracellular proteins stained as previously described [46]. Data were collected on a FACS Fortessa (BD) and analyzed with Stanford CytoBank software (http://cytobank.stanford.edu/public/openid/). Median fluorescence intensity (MFI) values were used as basis for data analyses. Antibodies used are listed in Table 2.

**Table 2:** Antibodies used in this study

**Antigen**	**Epitope**	**Clone**	**Conjugate**	**Manufacturer**
AKT	p-Ser473	F29-763	Alexa Fluor® 647	BD
CREB	p-Ser133	J151-21	Alexa Fluor® 647	BD
p38	p-Thr180/p-Tyr182	36/p38	Alexa Fluor® 488	BD
ERK1/2	p-Thr202/p-Tyr204	20A	Alexa Fluor® 488	BD
STAT1	p-Tyr701	4a	Alexa Fluor® 647	BD
STAT3	p-Tyr705	4/P-STAT3	Alexa Fluor® 488	BD
STAT5	p-Tyr694	47	Alexa Fluor® 647	BD
STAT6	p-Tyr641	18/P-STAT6	Alexa Fluor® 647	BD
p53	aa 1-45	DO-7	FITC	BD
p53	p-Ser37	J159-641.79	Alexa Fluor® 647	BD
p53	p-Ser15	16G8	Alexa Fluor® 488	Cell Signaling Technologies
p53	ac-Lys382	L82-51	Alexa Fluor® 647	BD
NF-κB	p-Ser529	K10-895.12.50	Alexa Fluor® 488	BD
c-Cbl	p-Tyr700	47/c-Cbl	Alexa Fluor® 488	BD
CD3	CD3	SK7	PE-Cy7	BD
CD20	CD20	H1(FB1)	PerCP-Cy 5.5	BD
CD163	CD163	Mac2-158	PE	Trillium Diagnostics

### Statistical and bioinformatical approaches

*Heatmaps*: Statistical comparisons were made using GraphPad PRISM (version 5.0, GraphPad Software, Inc., USA) software, with one-way analysis of variance (ANOVA) and Tukey’s multiple comparison post-tests to determine significant differences. A Student’s unpaired or paired t-test was employed when only two groups were compared. *Cluster analyses*: Analysis were performed using J-Express 2011 analysis suite (MolMine AS, Bergen, Norway) [47]. For each protein the mean fold change for the donors was calculated, for each leukocyte subset and each time point. Values were log(2) converted before unsupervised hierarchical clustering analyses were performed using Euclidean correlation with complete linkage.

## Competing interests

The authors declare that they have no competing interests.

## Authors’ contributions

TB and EE conceived, designed and performed experiments, analyzed data and wrote the manuscript. BSE participated during experiments and performed statistical analyses. AS participated during experiments and performed flow cytometric analyses. HR performed statistical analyses. HJA, ACJ, OKV and ØB conceived and guided the study. BTG conceived and guided the study, and wrote the manuscript. All authors read and approved the final manuscript.

## Pre-publication history

The pre-publication history for this paper can be accessed here:

http://www.biomedcentral.com/2050-6511/14/35/prepub

## Supplementary Material

Additional file 1: Table S1 Levels of significance for the observed in vitro induced phosphorylation/acetylation alterations of all target molecules. Click here for file

Additional file 2: Table S2 Levels of significance for the observed in vitro induced phosphorylation/acetylation alterations of p53. Click here for file
