# Supplementary material for: Distinct single cell signal transduction signatures in leukocyte subsets stimulated with khat extract, amphetamine-like cathinone, cathine or norephedrine
Source: BMC Pharmacol Toxicol. 2013 Jul 11;14:35. doi: 10.1186/2050-6511-14-35 (PMC3733921; doi:10.1186/2050-6511-14-35)
Supplement: Additional file 1: Table S1 — Levels of significance for the observed in vitro induced phosphorylation/acetylation alterations of all target molecules. [file 2050-6511-14-35-S1.doc]

|  |  | **Khat 3.16 x 10^-4^** | | | **Khat 10^-3^** | | | **Norephedrine 10^-4^ M** | | | **Cathine 10^-4^ M** | | | **Cathinone 10^-4^ M** | | | **Combination 10^-4^ M** | | |
| --- | --- | --- | --- | --- | --- | --- | --- | --- | --- | --- | --- | --- | --- | --- | --- | --- | --- | --- | --- |
|  |  | **4’** | **10’** | **15’** | **4’** | **10’** | **15’** | **4’** | **10’** | **15’** | **4’** | **10’** | **15’** | **4’** | **10’** | **15’** | **4’** | **10’** | **15’** |
| **T-lymphocytes** | p-STAT1 | ***** |  |  |  |  |  |  |  |  |  |  |  |  |  |  |  |  |  |
|  | p-c-Cbl |  |  |  |  | *** * *** |  |  |  |  | *** * *** | *** * *** | ***** |  |  |  | ***** |  |  |
|  | p-ERK1/2 |  |  |  | ***** | *** * *** |  |  |  |  | *** * *** | *** * *** | ***** |  |  |  |  |  |  |
|  | p-STAT3 |  |  |  |  |  |  |  |  |  |  |  |  |  |  |  |  |  |  |
|  | p-NF-kB |  |  |  |  |  |  |  |  |  |  |  |  |  |  |  |  |  |  |
|  | p-p38 MAPK |  | *** *** | *** * *** | *** * *** | *** * *** | *** * *** |  |  |  | *** * *** | *** *** |  |  |  |  |  |  |  |
|  | p-STAT6 | *** * *** |  | ***** |  |  |  |  |  |  |  |  |  |  |  |  |  |  |  |
|  | p-AKT | *** * *** |  |  |  |  |  |  |  |  |  |  |  |  |  |  |  |  |  |
|  | p53 total |  |  |  | ***** | *** * *** |  |  |  |  | ***** |  |  |  |  |  |  |  |  |
|  | p53 p-Ser15 |  |  |  | ***** | *** * *** |  |  |  |  | *** * *** | *** * *** | ***** |  |  |  | ***** |  |  |
|  | p53 p-Ser37 |  |  |  |  |  |  |  |  |  |  |  |  |  |  |  |  |  |  |
|  | p53 ac-Lys382 |  |  |  |  |  |  |  |  |  |  |  |  |  |  |  |  |  |  |
| **B-lymphocytes** | p-STAT1 | ******* |  |  | ***** |  |  |  |  |  |  |  |  |  |  |  |  |  |  |
|  | p-c-Cbl |  |  |  |  | *** * *** |  |  |  |  | *** * *** | *** * *** | *** *** |  |  |  |  |  |  |
|  | p-ERK1/2 |  |  |  | ***** | *** *** |  |  |  |  | *** *** | *** * *** | *** *** |  |  |  |  |  |  |
|  | p-STAT3 |  |  |  | ***** | *** * *** | ***** |  |  |  |  |  |  |  |  |  |  |  |  |
|  | p-NF-kB |  |  |  | ***** | ***** |  |  |  |  |  |  |  |  |  |  |  |  |  |
|  | p-p38 MAPK |  |  | ***** | *** * *** | *** * *** | *** *** |  |  |  | *** * *** | *** * *** |  |  |  |  |  |  |  |
|  | p-STAT6 | *** * *** |  |  |  |  |  |  |  |  |  |  |  |  |  |  |  |  |  |
|  | p-AKT | *** * *** |  | ***** |  |  |  |  |  |  |  |  |  |  |  |  |  |  |  |
|  | p53 total |  |  |  | *** *** | *** * *** | ***** |  |  |  | *** * *** |  |  |  |  |  |  |  |  |
|  | p53 p-Ser15 |  |  |  |  | *** * *** |  |  |  |  | *** * *** | *** * *** | ***** |  |  |  | ***** |  |  |
|  | p53 p-Ser37 |  | ***** |  |  |  |  |  |  |  |  |  |  |  |  |  |  |  |  |
|  | p53 ac-Lys382 |  |  |  |  |  |  |  |  |  |  |  |  |  |  |  |  |  |  |
| **NK-cells** | p-STAT1 | *** * *** |  |  |  |  |  |  |  |  |  |  |  |  |  |  |  |  |  |
|  | p-c-Cbl |  |  |  | ***** | *** * *** |  |  |  |  | *** * *** | *** * *** |  |  |  |  |  |  |  |
|  | p-ERK1/2 |  |  |  | *** * *** | *** * *** | ***** |  |  |  | *** *** | *** * *** |  |  |  |  |  |  |  |
|  | p-STAT3 |  |  | ***** | *** * *** | *** * *** | *** *** | ***** |  |  |  |  |  |  |  |  |  |  |  |
|  | p-NF-kB |  |  | ***** | ***** | *** *** | *** *** |  |  |  |  |  |  |  |  |  |  |  |  |
|  | p-p38 MAPK |  |  |  | *** * *** | *** * *** | *** * *** |  |  |  | *** *** | *** *** |  |  |  |  |  |  |  |
|  | p-STAT6 | *** * *** |  |  |  |  |  |  |  |  |  |  |  |  |  |  |  |  |  |
|  | p-AKT | *** * *** |  |  |  |  |  |  |  |  |  |  |  |  |  |  |  |  |  |
|  | p53 total |  |  | ***** | *** *** | *** * *** | *** *** |  |  |  |  |  |  |  |  |  |  |  |  |
|  | p53 p-Ser15 |  |  |  | *** *** | *** * *** | ***** |  |  |  | *** * *** | *** * *** |  |  |  |  |  |  |  |
|  | p53 p-Ser37 |  |  |  |  |  |  |  |  |  |  |  |  |  |  |  |  |  |  |
|  | p53 ac-Lys382 |  |  |  |  |  |  |  |  |  |  |  |  |  |  |  |  |  |  |
| **Monocytes** | p-STAT1 | ***** |  |  |  |  |  |  |  |  |  |  |  |  |  |  |  |  |  |
|  | p-c-Cbl |  |  |  | ***** | *** *** |  |  |  |  | *** *** | ***** |  |  |  |  |  |  |  |
|  | p-ERK1/2 |  |  |  | ***** | *** *** |  |  |  |  |  |  |  |  |  |  |  |  |  |
|  | p-STAT3 |  |  |  |  |  |  |  |  |  |  |  |  |  |  |  |  |  |  |
|  | p-NF-kB |  |  |  |  |  |  |  |  |  |  |  |  |  |  |  |  |  |  |
|  | p-p38 MAPK | *** * *** | *** *** | *** * *** | *** * *** | *** * *** | *** * *** |  |  |  |  |  |  |  |  |  |  |  |  |
|  | p-STAT6 | *** * *** |  |  |  |  |  |  |  |  |  |  |  |  |  |  |  |  |  |
|  | p-AKT | *** * *** |  |  |  |  |  |  |  |  |  |  |  |  |  |  |  |  |  |
|  | p53 total |  |  |  | ***** | *** *** |  |  |  |  |  |  |  |  |  |  |  |  |  |
|  | p53 p-Ser15 |  |  |  | *** *** | *** *** |  |  |  |  | *** * *** |  |  |  |  |  |  |  |  |
|  | p53 p-Ser37 |  |  |  |  |  |  |  |  |  |  |  |  |  |  |  |  |  |  |
|  | p53 ac-Lys382 |  |  |  |  |  |  |  |  |  |  |  |  |  |  |  |  |  |  |

Supplementary table 1. Green colour indicates a significant increase in phosphorylation/acetylation, purple colour indicates a significant reduction. * denotes p < 0.05, * * denotes p < 0.001, * * * denotes p < 0.0001, ’ denotes minutes.
