# Supplementary material for: Distinct single cell signal transduction signatures in leukocyte subsets stimulated with khat extract, amphetamine-like cathinone, cathine or norephedrine
Source: BMC Pharmacol Toxicol. 2013 Jul 11;14:35. doi: 10.1186/2050-6511-14-35 (PMC3733921; doi:10.1186/2050-6511-14-35)
Supplement: Additional file 2: Table S2 — Levels of significance for the observed in vitro induced phosphorylation/acetylation alterations of p53. [file 2050-6511-14-35-S2.doc]

|  |  | **Khat 3.16 x 10^-4^** | | | **Khat 10^-3^** | | | **Noreph. 10^-4^ M** | | | **Cathine 10^-4^ M** | | | **Cathinone 10^-4^ M** | | | **Comb. 10^-4^ M** | | |
| --- | --- | --- | --- | --- | --- | --- | --- | --- | --- | --- | --- | --- | --- | --- | --- | --- | --- | --- | --- |
|  |  | **4’** | **10’** | **15’** | **4’** | **10’** | **15’** | **4’** | **10’** | **15’** | **4’** | **10’** | **15’** | **4’** | **10’** | **15’** | **4’** | **10’** | **15’** |
| **T-lymphocytes** | p53 p-Ser15 |  |  |  |  |  |  |  |  |  | * * * | * * * | * * * | * | * * |  | * * | * * | * |
|  | p53 p-Ser37 |  |  |  |  | * |  |  |  |  |  |  |  |  |  |  |  |  |  |
|  | p53 ac-Lys382 |  |  |  |  |  |  |  |  |  |  |  |  |  |  |  |  |  |  |
| **B-lymphocytes** | p53 p-Ser15 |  |  |  |  |  |  |  |  |  | * * | * * * | * * |  | * * * | * | * * | * * | * * |
|  | p53 p-Ser37 |  |  |  |  |  |  |  |  |  |  | * | * |  |  |  |  |  |  |
|  | p53 ac-Lys382 |  |  |  |  |  |  |  |  |  |  |  |  |  |  |  |  |  |  |
| **NK-cells** | p53 p-Ser15 |  |  |  |  |  |  |  |  |  | * * * | * * * | * * |  | * * |  | * * | * * |  |
|  | p53 p-Ser37 |  |  |  |  | * |  |  |  |  |  |  |  |  |  |  |  |  |  |
|  | p53 ac-Lys382 |  |  |  |  | * |  |  |  |  |  |  |  |  |  |  |  |  |  |
| **Monocytes** | p53 p-Ser15 |  |  |  |  |  |  |  |  |  | * * |  |  |  |  |  |  |  |  |
|  | p53 p-Ser37 |  |  |  |  |  |  |  |  |  |  |  |  |  |  |  |  |  |  |
|  | p53 ac-Lys382 |  |  |  |  | * |  |  |  |  |  |  |  |  |  |  |  |  |  |

Supplementary table 2. Green colour indicates a significant increase on phosphorylation/acetylation, and purple colour indicates

a significant reduction. * denotes p < 0.05, * * denotes p < 0.001, * * * denotes p < 0.0001, ’ denotes minutes.
